# Influencing Factors of International Students’ Anxiety Under Online Learning During the COVID-19 Pandemic: A Cross-Sectional Study of 1,090 Chinese International Students

**DOI:** 10.3389/fpsyg.2022.860289

**Published:** 2022-04-14

**Authors:** Yejun Tan, Zhijian Wu, Xiangnan Qu, Yuzhuo Liu, Lele Peng, Yan Ge, Shu Li, Jinfeng Du, Qi Tang, Jia Wang, Xiaofei Peng, Jiafen Liao, Meiyan Song, Jin Kang

**Affiliations:** ^1^Department of Rheumatology and Immunology, The Second Xiangya Hospital of Central South University, Changsha, China; ^2^School of Mathematics, University of Minnesota Twin Cities, Minneapolis, MN, United States; ^3^Department of Cardiovascular Medicine, The Second Xiangya Hospital of Central South University, Changsha, China; ^4^Xiangya School of Medicine, Changsha, China; ^5^Department of Psychiatry, The Second Xiangya Hospital of Central South University, Changsha, China; ^6^Department of Endocrinology, Wangwang Hospital of Hunan, Changsha, China

**Keywords:** international students, anxiety, COVID-19, online learning, public health emergency, mental health, online survey, cross-sectional study

## Abstract

**Objective:**

We conducted the following cross-sectional study to comprehensively assess the anxiety among Chinese international students who studied online during the COVID-19 pandemic and its influencing factors.

**Methods:**

Questionnaires were distributed through “Sojump,” and a total of 1,090 valid questionnaires were collected. The questionnaire was divided into two parts: general situation and anxiety assessment of students. The former used a self-made questionnaire, and the international general GAD-7 scale was used to measure anxiety. Chi-square test was used to analyze the differences between groups, and logistic regression analysis was performed for the factors with differences.

**Results:**

Anxiety was found in 707 (64.9%) of 1,090 international students. Chi-square test and multivariate Logistic regression analysis showed that the incidence of anxiety was higher in the group under 22 years of age than in the group over 22 years of age (68% vs. 61%, *p* = 0.015; OR = 1.186, 95% CI 1.045–1.347, *p* = 0.008); International students living in big cities had a higher incidence of anxiety than those living in rural areas (67% vs. 60%, *p* = 0.022; OR = 1.419, 95%CI 1.038–1.859, *p* = 0.011); international students who socialized 3 times or less monthly had a higher incidence of anxiety than those who socialized more than 3 times per month (68% vs. 58%, *p* = 0.003; OR = 1.52, 95%CI 1.160–1.992, *p* = 0.002); international students who expected purely online teaching had a higher incidence of anxiety than those who expected purely offline teaching or dual-track teaching (72% vs. 64%, *p* = 0.037; OR = 1.525, 95%CI 1.069–2.177, *p* = 0.02); international students with a subjective score of online learning experience of 6 or less had a higher incidence of anxiety than those with subjective scores of more than 6 (70% vs. 60%, *p* = 0.001, OR = 1.25, 95%CI 1.099–1.422, *p* = 0.001). However, gender, emotional status, BMI, major of study, vaccination status, and degree type had no significant difference in the incidence of anxiety among international students who studied online during the COVID-19 pandemic.

**Conclusion:**

During COVID-19, international students who were younger, came from big cities, had low social frequency, expected purely online teaching, and had poor experience of online classes were risk factors for anxiety during online classes.

## Introduction

In January 2020, the World Health Organization listed COVID-19 as a public health emergency of international concern (Yue et al., 2020). As of 2 August 2021, COVID-19 has rapidly spread to 208 countries and territories, making it a global pandemic with over 200 million infections and over 4 million deaths worldwide. Studies have shown that COVID-19 has caused a psychological crisis in the public that urgently needs to be addressed (Choi et al., 2020; Dong and Bouey, 2020; Gao et al., 2020; Gómez-Salgado et al., 2020; Huang and Zhao, 2020; Wang et al., 2020; Yang et al., 2020). The COVID-19 pandemic has triggered a psychological crisis among the public because of the increased prevalence of mental illness, including anxiety and depression (Asmundson and Taylor, 2020; González-Sanguino et al., 2020; Kola, 2020; Ozamiz-Etxebarria et al., 2020; Ahn et al., 2021). The public’s positive psychological response to COVID-19 has played a crucial role in reducing anxiety. Positive thoughts and attitudes can help individuals cope with stressors (Görgen et al., 2014). A recent study found that the hope helps prevent anxiety (Mirhosseini et al., 2020). The theory of rational emotive behavioral therapy suggests that rational beliefs can relieve anxiety and other symptoms of mental distress (David et al., 2018; Eseadi, 2019). In addition, cognitive-behavioral models of health anxiety suggest that negative emotions and misunderstanding of health-related stimuli may increase the chance of developing anxiety (Gautreau et al., 2015; Hagger et al., 2017; Schäfer et al., 2017). Therefore, promoting positive psychological response, initiating emotional regulation, and positive cognition of health-related information, such as maintaining a positive attitude and rationality, is necessary for the public to better cope with stress (Grecucci et al., 2015). It is worth noting that the psychological impact of COVID-19 on students is significant. Previous studies have also shown that after the government blockaded the city for a week, the rating of anxiety and depression among students increased significantly and reached a stable state in the second week but remained at a poor level (Jin and Fung, 2021). Two months after online learning, the anxiety and depression among students still increased significantly (Magson et al., 2021). So, after implementing online courses for a year, what is the students’ psychological state? However, no relevant literature has been reported so far.

International students, primarily college students, are a huge and leading group for world cultural and academic exchanges. According to the Ministry of Education, PRC, there were 700,000 Chinese students studying abroad in 2019. With the increasing demand for academic qualifications in society, international students have increased yearly. The mental health of this particular group has attracted more and more attention (Wang et al., 2015).

To avoid infection or implement border blockade policies, many schools worldwide have transferred traditional offline teaching to online. The outbreak of COVID-19 has also forced many Chinese international students to stay in China for online learning. When the teaching mode changed from the expected foreign campus to the computer at home, such large-scale online teaching came as suddenly as COVID-19, international students were significantly affected both physically and mentally. Therefore, as a particular group, the anxiety and its influencing factors of international students under online learning during the COVID-19 pandemic are worthy of our attention. However, for example, previous studies have not focused on issues of mental health among international students or the context of online learning among international students during the COVID-19 pandemic. To better understand the anxiety among international students under online learning and its influencing factors during the COVID-19 pandemic, we conducted a cross-sectional study on the anxiety and its influencing factors of international students.

The main objective of this study was to assess anxiety and its influencing factors in a sample of 1,090 Chinese international students who studied online during the outbreak of COVD-19. In this study, A standardized mental health measure (GAD-7) was used to assess acute anxiety symptoms. Our study found that age, cities of residence, the frequency of socialization, expected teaching mode, and subjective experience of online learning influenced the anxiety of international students.

## Materials and Methods

### Research Object and Procedures

To investigate the anxiety among international students who studied online during the COVD-19 pandemic, we adopted a cross-sectional study design and questionnaires through the “Sojump,” an Internet-based questionnaire application in mainland China. We distributed questionnaires on the platform of 15 WeChat international student groups with a total of about 15*500 = 7,500 people. This survey adopted the principle of voluntary participation, and 1,101 questionnaires were collected. 11 questionnaires were invalid for interfering with common sense problems, and 0 were excluded within 2 min. A total of 1,090 valid questionnaires were collected, and the recovery rate was 99%. The WeChat group of international students was established spontaneously by Chinese international students to facilitate communication in all aspects of study and life abroad. The person in charge of this survey is in charge of the WeChat group mentioned above. During the outbreak of COVID-19, we distributed questionnaires in the name of the person in charge to understand the psychological conditions of the students during online learning.

The survey was conducted from August 15 to 25, 2021, during which we obtained a sufficient sample size in accordance with previous related studies (Hu et al., 2007; Kamangar and Islami, 2013; Zhang et al., 2017; Liu et al., 2020). The questionnaire consisted of two parts: the internationally recognized Chinese Version of the Generalized Anxiety Disorder Scale (GAD-7) and the self-designed general situation of international Students who studied online due to the outbreak of the COVID-19 pandemic. It takes about 3–5 min to complete the questionnaire. The inclusion criteria for participating in this study were: international students who have taken online courses for a certain period, have Chinese reading and writing ability, can use smartphones to complete the questionnaire, and were willing to participate. Exclusion criteria were any treatment for mental illness, any history of drug dependence, and any diagnosis of illness or injury that might prevent them from completing the questionnaire independently.

The questionnaire was submitted after the participants had answered all the questions. Only data from the complete questionnaires were analyzed.

### Moral Statement

The study was carried out by the Declaration of Helsinki (1989). This study was approved by the Ethics Committee of Xiangya Hospital of Central South University. In the process of informed consent in the preamble of the questionnaire, we left the contact information of the project team members for the respondents. If the international students need professional psychological consultation, they can contact us through our contact information.

### Demographics and the General Situation of International Students Who Studied Online During the COVID-19 Pandemic

Demographic characteristics of participants were collected in this study, including gender, age, emotional status, BMI, city of residence, the location of the school they attended, major of study, vaccination status, and degree type. During the COVID-19 pandemic, the general situation of international students who studied online included advantages and disadvantages of online learning, time difference, average daily study duration, average daily sleep duration, the frequency of physical exercise, the frequency of socialization, primary ways to relieve stress, measures to deal with infection risk, expected teaching modes in coming semesters, concerns about COVID-19 infection, and subjective ratings of the online learning experience. The significant advantages and disadvantages of online learning, the primary ways to relieve stress, and the primary measures to deal with the risk of infection were multiple-choice questions. Participants chose at least one of these multiple choices and could choose up to five. The subjective score of online learning experience during the COVID-19 pandemic was 10 points, with 0 points for completely dissatisfied and 10 points for completely satisfied.

### Measurement of Anxiety

Gad-7 is one of the most reliable measures of generalized anxiety disorder. Anxiety-related psychological problems were assessed on the Likert-4 scale, with options ranging from “totally uncertain = 0,” “a few days =1,” “more than half the time = 2,” and “almost every day =3” on a scale of 0 to 21. The threshold of anxiety was set to higher than 5 based on the Gad-7 score (Ahn et al., 2021; Park et al., 2021; Yoo et al., 2021).

### Statistical Analysis

SPSS software (version 23.0) used to perform all statistical analysis. The Chi-square test was used to analyze the difference in anxiety degree among groups. Anxiety was assessed using a binary variable (anxiety or non-anxiety, measured on the GAD-7 scale). The variables included in multivariate logistic regression analysis included age, city of residence in China, subjective experience score of online learning, the frequency of socialization, and expected teaching method, all of which were two variables. When the two-tail *p*-value was less than 0.05, the result was considered to be statistically significant.

## Results

### Demographic Characteristics and Prevalence of Anxiety Among International Students Who Studied Online During the COVID-19 Pandemic

Our study found anxiety in 707 (64.9%) of 1,090 international students. 1,101 international students participated in the survey, of which 1,090 were valid questionnaires with an effective rate of 99.0%. *The general situation of international students and their opinions toward online learning are shown in* Table 1. Students from schools in Europe and Hong Kong, Macao, and Taiwan accounted for 37.8 and 27.1%, respectively, while those from schools in Oceania accounted for less than 10%(6.3%). The top three majors chosen by international students were economics and finance, management, and engineering, accounting for 21, 15, and 12%, respectively. History, agriculture, and military science were the least popular, accounting for less than 1%. The top three advantages of online learning were effectively reducing the risk of COVID-19 infection, saving living and commuting expenses, and saving commuting time, with a cumulative selection rate of 80, 69, and 69%, respectively. The two significant disadvantages of online learning were the difficulty of concentration and the lack of campus atmosphere of studying abroad. The cumulative selection rate was 70 and 68%, respectively. In addition, primary ways for international students to release pressure were watching TV series & movies (77%) and chatting with friends online (66%), followed by dining out & shopping (49%) and physical exercise (44%). The primary measures for these international students to deal with the risk of infection were wearing masks (89%), getting vaccinated (82%), maintaining social distance (76%), washing hands frequently (74%), and reducing the times of visiting crowded places (71%), while only 11% of them chose to drop out (3%) or transfer to another school (8%).

**Table 1:** General situation of 1,090 Chinese international students who studied online during the COVID-19 pandemic.

Variables	Frequency	Chi-square	*p*-value
The location of schools			
North America	151(13.9)	1945.68	0
Europe	412(37.8)		
Asia	163(15)		
Oceania	69(6.3)		
Hong Kong, Macao and Taiwan	295(27.1)		
Major of study			
Philosophy	13(0.01)	702.04	0
Economics and finance	230(0.21)		
Law	39(0.04)		
Pedagogy	122(0.11)		
Literature	123(0.11)		
History	12(0.01)		
Science	101(0.09)		
Engineering	133(0.12)		
Agronomy	10(0.01)		
Medicine	61(0.06)		
Management	166(0.15)		
Art	79(0.07)		
Military science	1(0)		
Advantages of online learning			
Saving living and commuting expenses	747(0.69)	1369.86	0
Saving commuting time	751(0.69)		
Effectively reduce the risk of COVID-19 infection	872(0.8)		
No extra time needed to get used to the new environment	387(0.36)		
No social fear	329(0.3)		
Online courses can be played repeatedly for easy understanding and review	582(0.53)		
Online courses are fast paced and more efficient	130(0.12)		
Translation software/plug-ins can be used to solve the language barrier	296(0.27)		
Online courses may have higher grades	101(0.09)		
Disadvantages of online learning			
Time difference can easily lead to a chaotic daily routine	337(0.31)	918.7	0
The Difficulty of concentration	744(0.68)		
Poor network connection	557(0.51)		
Unable to interact with teachers immediately	552(0.51)		
Inconvenience to exchange ideas with classmates	560(0.51)		
The lack of campus atmosphere of studying abroad	768(0.7)		
Lack of social interaction	312(0.29)		
Inconvenience for scientific research	189(0.17)		
The trouble of Preparing for exams	129(0.12)		
The ways to relieve stress			
Chatting with friends online	719(0.66)		
Watching TV series& movies	841(0.77)		
Shopping and dining out	539(0.49)		
Doing physical exercise	480(0.44)		
Playing video games	377(0.35)		
Massage	125(0.11)		
Watching ASMR videos	96(0.09)		
Primary measures to address the risk of COVID-19 infection			
Vaccination	894(0.82)	1922.38	0
Wearing a mask	971(0.89)		
Washing your hands regularly	806(0.74)		
Keeping Social distance	832(0.76)		
Avoid visiting crowded places	772(0.71)		
Gap year/delay until the epidemic stabilizes	190(0.17)		
Drop out of school	33(0.03)		
Transfer to another schools in areas where the epidemic is stable	83(0.08)		

### Factors Influencing the Anxiety Among International Students Who Studied Online During the COVD-19 Pandemic

Students with GAD-7 scores higher than or equal to 5 were defined as the anxiety group, and the Chi-square test was used to analyze the influencing factors of anxiety among the international students who studied online due to the outbreak of COVD-19. The results are shown in Table 2. Among the international students who studied online, the incidence of anxiety was 68%(383/561) among the international students under the age of 22, compared with 61%(324/529) among those higher or equal to the age of 22. There was a statistical significance between the two groups (Chi-square = 5.893, *p = 0.015*). Among the international students who studied online, 67%(500/745) of those students living in first-tier and provincial capital cities and Hong Kong, Macao, and Taiwan *(hereinafter referred to as big cities)* felt anxious. In comparison, 60%(207/345) of international students living outside first-tier and provincial capital cities and in rural areas *(hereinafter referred to as small cities and rural areas)* felt anxious. There was a statistical significance between the two groups (Chi-square = 5.236, *p = 0.02*). Among the international students who studied online, 68%(512/756) of them who socialized three times or less monthly felt anxious about online learning, compared with 58%(195/334) of students who socialized three times or more monthly. There was a statistical significance between the two groups (Chi-square = 8.87, *p = 0.003*). Among the international students who studied online, the incidence of anxiety among those who wished for purely offline teaching or dual-track online and offline teaching was 64%(567/907), compared with 72%(131/183) among those who wished purely online teaching. There was a statistical significance between the two groups (Chi-square = 4.361, *p = 0.037*). Among the international students who studied online, 70%(341/484) students with a subjective score of online learning experience during the COVID-19 pandemic of 6 or less felt anxious, while 60%(366/696) of them with a subjective score of more than 6 felt anxious. There was a statistical significance between the two groups (Chi-square = 11.945, *p = 0.001*). However, gender, emotional status, BMI, vaccination or not, degree type, time difference, daily study duration, daily sleep duration, and frequency of physical exercise had no significant difference in the incidence of anxiety among international students who studied online.

**Table 2:** Analysis of influencing factors on the incidence of anxiety during online courses for international students.

	Non-anxiety n (%)	Anxiety n (%)	Total	Chi-square	*p*-value
Gender					
Male	95(0.37)	160(0.63)	255	0.665	0.48
Female	288(0.34)	547(0.66)	835		
Age					
≥22 years old	178(0.32)	383(0.68)	561	5.893	0.02
<22 years old	205(0.39)	324(0.61)	529		
Relationship status					
Single	256(0.34)	500(0.66)	756	1.760	0.19
Non-single	127(0.38)	207(0.62)	334		
BMI					
Low	103(0.31)	230(0.69)	333	3.734	0.16
Medium	242(0.37)	411(0.63)	653		
Height	38(0.37)	66(0.63)	104		
City of residence in China					
Big cities	245(0.33)	500(0.67)	745	5.236	0.02
Small cities and rural areas	138(0.4)	207(0.6)	345		
COVID-19 vaccination status					
Completed vaccination	306(0.35)	572(0.65)	878	0.162	0.69
Incomplete vaccination	77(0.36)	135(0.64)	212		
Degree type					
Undergraduate and below	166(0.34)	318(0.66)	484	0.270	0.60
Postgraduate and above	217(0.36)	389(0.64)	606		
Time difference in online courses					
With time difference	121(0.33)	250(0.67)	371	1.571	0.20
Without time difference	262(0.36)	457(0.64)	719		
The average daily study duration					
≤5 h	236(0.35)	430(0.65)	666	0.067	0.80
>5 h	147(0.35)	277(0.65)	424		
The average daily sleep duration					
≤6 h	54(0.33)	111(0.67)	165	3.391	0.18
6–8 h	221(0.34)	432(0.66)	653		
>8 h	108(0.4)	164(0.6)	272		
Frequency of physical exercise					
No physical exercise	98(0.32)	210(0.68)	308		
1–2 times weekly	178(0.37)	308(0.63)	486	2.094	0.35
>3 times weekly	107(0.36)	189(0.64)	296		
Frequency of socialization (e.g., shopping, eating, and going to the movies)					
≤3 times monthly	244(0.32)	512(0.68)	756	8.870	0.00
>3 times monthly	139(0.42)	195(0.58)	334		
The expected teaching mode in coming semesters (2021 fall & 2022 spring)					
Pure *offline* teaching/*online and offline dual-track* teaching	331(0.36)	576(0.64)	907	4.361	0.04
Pure *online* teaching	52(0.28)	131(0.72)	183		
The concerns about the risk after forcing mandatory on-campus teaching					
Worry	341(0.34)	652(0.66)	993	3.112	0.08
No worry	42(0.43)	55(0.57)	97		
Subjective score of the experience of online learning during the COVID-19 pandemic (from 1 to 10)					
≤6	143(0.3)	341(0.7)	484	11.945	0.00
>6	240(0.4)	366(0.6)	606		

### Logistic Analysis of Factors Influencing the Incidence of Anxiety Among International Students Who Studied Online During the COVID-19 Pandemic

Our study combined age, the resident city in China, the frequency of socialization, expected teaching methods, and subjective experience score of online courses to construct a multi-factor Logistic regression equation (Table 3). We found that international students under the age of 22 were at increased risk of anxiety compared with those higher or equal to the age of 22, and the difference was statistically significant (OR = 1.186, 95%CI 1.045–1.347, *p = 0.008*). Compared with international students in small cities and rural areas, the students living in big cities were at increased risk of anxiety, and the difference was statistically significant (OR = 1.419, 95%CI 1.038–1.859, *p = 0.01*). Compared with the international students with a subjective score of online learning experience during the COVID-19 pandemic higher than 6, students with a subjective score less than or equal to 6 were at increased risk of anxiety, and the difference was statistically significant (OR = 1.25, 95%CI 1.099–1.422, *p = 0.001*). Compared with the international students who socialized more than three times a month, those who socialized less than three times per month were at increased risk of anxiety, and the difference was statistically significant (OR = 1.52, 95%CI 1.160–1.992, *p = 0.002*). Compared with the international students who wished for purely offline or online and offline dual-track teaching in coming semesters, those who wished for purely online teaching were at increased risk of anxiety, and the difference was statistically significant (OR = 1.525, 95%CI 1.069–2.177, *p = 0.02*).

**Table 3:** Logistic regression analysis of the incidence of anxiety during online courses for international students.

		Wald	*p*-value	OR-value	95% confidence	
	B					
		Chi-square value			Interval	
Age						
≥22 years of age^*^	0.171	6.931	0.008	1.186	1.045	1.347
<22 years of age						
City of residence						
Small cities and rural areas^*^	0.35	6.449	0.01	1.419	1.083	1.859
Big cities						
Subjective score of the online learning (from 1 to 10)						
>6^*^	0.223	11.52	0.001	1.25	1.099	1.422
≤6						
Frequency of socialization						
>3 times monthly^*^	0.419	9.204	0.002	1.52	1.16	1.992
≤3 times monthly						
The expected teaching mode in coming semesters						
*Offline* teaching or *online* and *offline* dual-track teaching^*^	0.422	5.414	0.02	1.525	1.069	2.177
Pure *online* teaching						

^*^Is the control group.

## Discussion

With the development of globalization and increasingly fierce competition in society, international students are increasing year by year. As a result, the mental health of this unique group is also attracting more and more attention. Previous studies have suggested that international students in higher education are prone to mental health problems, such as depression and anxiety (Wang et al., 2015). At the same time, international students are the vulnerable group with apparent mental health problems, which indicates that the research on international students’ mental health is rapidly expanding and developing toward a new research direction (Han et al., 2013; Wang et al., 2015). We observed that 707 out of 1,090 Chinese international students participating in the study felt anxious, accounting for 64.86%. The prevalence of these international students significantly exceeds the post-pandemic incidence of anxiety among the general population (31.9%; Salari et al., 2020). Apparently, the prevalence of anxiety among these international students was twice that of the general population. Since universities in many countries have been forced to switch from offline to online teaching due to the high contagiousness of COVID-19, international students were forced to return to their home countries for online study. The mode of online teaching can be a challenge for many international students. This survey was conducted 1 year after the outbreak of COVID-19 in China, mainly to understand the situation of international students from China after 1 year of online learning and the influencing factors of anxiety.

Our research showed that international students from schools in Europe (37.8%) and Hong Kong, Macao, and Taiwan (27.1%) accounted for the majority, while those from schools in Oceania accounted for less than 10% (6.3%). From the perspective of the regions chosen by international students, fewer international students chose schools in Oceania. Their choices may be affected by the border blockade policies of Oceanian countries, such as Australia and New Zealand. As a result, many international students can only study online in China for a long time and therefore do not consider Oceania as their first choice. In addition, our results showed that the proportion of international students who chose to study in Asia (Hong Kong, Macau, Taiwan, and other parts of Asia) was as high as 42.1%. It is not hard to see that Asia has become one of the new education centers, attracting more international students. According to our results, the top three majors chosen by international students were economics, management, and engineering, accounting for 21, 15, and 12%, respectively. History, agriculture, and military sciences, by contrast, accounted for less than 1%. The distribution of majors chosen by international students is relatively consistent with the popular majors chosen by Chinese college students (Wu et al., 2021).

The top three advantages of online learning were effectively reducing the risk of COVID-19 infection, saving living and commuting expenses, and saving commuting time, with the cumulative selection rate of 80, 69, and 69%, respectively. Online learning, where students are primarily at home, can effectively reduce the probability of COVID-19 infection by avoiding gatherings (Wu et al., 2021). At the same time, since the price of necessities in Europe and the United States is much higher than that in mainland China, students can save a lot expenses. The two most significant disadvantages of online learning were the difficulty of concentration and the lack of campus atmosphere of studying abroad, with a cumulative selection rate of 70 and 68%, respectively. It can be seen that online courses without face-to-face supervision by teachers and the atmosphere of studying together with other students is likely to cause distraction in class, which is consistent with the research of Mukhtar et al. (2020). This is why online learning places higher demands on self-discipline of students. At the same time, online learning, as the scope of activity for international students is mainly at home, international students who were unable to experience campus life have become the regret of those who studied online during the COVID-19 pandemic.

Nevertheless, international students can make up for this by interacting with their peers in China. Gradually, with the control of COVID-19 and the resumption of offline classes, It is believed that these international students taking online courses during the COVID-19 pandemic will cherish campus life more after returning to campus. Therefore, in the long run, short-term online learning may play a positive role in promoting international students to experience the campus culture and atmosphere.

Watching TV series & movies and chatting with friends accounted for 77 and 66%, respectively. However, the proportion of dining out & shopping and physical exercise was only 49 and 44%, respectively. According to our study, international students tended to choose leisure activities that can be completed at home and on the Internet, such as watching TV series and movies and chatting with friends online, while those that need to be completed outdoors, such as dining out and shopping and physical exercise were relatively low. On the one hand, affected by the COVID-19, students may purposely avoid gathering. On the other hand, most students may like to stay alone. With the COVID-19 pandemic under control, students can subjectively increase some recreational ways to interact with others.

The top five measures to deal with risk of COVID-19 infection were wearing a mask, getting vaccinated, maintaining social distance, washing hands frequently, and reducing visits to crowded places (89, 82, 76, 74, and 71%, respectively). It is not hard to see that more than a year after the outbreak of COVID-19, with the exception of vaccination, other anti-epidemic measures were consistent with the measures taken at the beginning of the COVID-19 outbreak (Liguori and Winkler, 2020). At the same time, the results of this study are consistent with the results of another study on Chinese college students (Shen et al., 2021). Combining the two studies, it can be concluded that Chinese college students, whether studying in China or abroad, have high support and compliance for non-drug intervention (NPI) prevention.

Our research (Table 2) showed that age, geography, the frequency of socialization, expected teaching methods, and subjective experience of online learning were influencing factors for international students’ anxiety. These related factors were also the core of this study, which can provide part of the basis for colleges and universities to quickly screen international students with a high risk of anxiety. Colleges and universities can carry out early intervention for students with risk through health promotion and psychological education so that the limited psychological consultation resources can reach the students who are most likely to benefit and then reduce the occurrence of anxiety among international students (Gladstone et al., 2021).

### Age Factors

Our findings suggested that age was strongly associated with positive psychological responses. The prevalence of anxiety was higher among the international students under the age of 22 (68%) than those higher or equal to the age of 22 (61%), suggesting that younger age may be a risk factor for anxiety among these international students who studied online during the COVID-19 pandemic. Our result is consistent with previous studies showing that younger age is a risk factor for anxiety (Brenes, 2006; Guo et al., 2016; Xiong et al., 2020). Compared with older students, young international students have less social cognition and future prediction because young international students tend to have less social practice and experience. With lower self-regulation and psychological resilience, the younger group tends to be worried about the uncertain future and negative academic impact of online teaching in the context of COVID-19, such as the acceptance of online courses by future employers. Conversely, due to their rich social experience and cognition, international students in the older group have stronger psychological endurance. As a result, they are less worried about negative impacts. Therefore, schools should pay more attention to young international students or carry out psychological counseling to relieve the anxiety of younger international students.

### Geographical Factors

Our study also found that geographical factors were an influencing factor for anxiety. Previous studies have pointed to a higher prevalence of anxiety disorders in the urban area of China (7.6%) than in rural China (4.66%; Guo et al., 2016). Our result is also in line with the high incidence of anxiety disorders in cities during the COVID-19 pandemic (Ren et al., 2020). Living in big cities was a risk factor for anxiety for international students who studied online during the COVID-19 pandemic. In addition, according to our survey data, most of these international students (745/1090) came from big cities, which is also related to the developed economy, high education level, and active thinking. This phenomenon reflects that the distribution of educational resources in China is relatively concentrated in big cities. International students from big cities live in more developed regions, where talent concentration leads to greater competition, thus increasing risk factors for anxiety.

Moreover, the cost of living in big cities, such as housing prices, is higher than in relatively small cities and rural areas, which may also increase the stress of international students. Faced with the pressure from fierce competition and high living costs, these international students who studied online in big cities may worry about the lack of competitiveness caused by online learning or other factors, thus increasing the risk of anxiety. Therefore, schools and health professionals should pay more attention to the mental health of international students from big cities.

### The Frequency of Socialization

In this study, the frequency with which international students socialized with others (such as shopping and dining out and watching movies) 3 times or less a month was a risk factor for anxiety. Among international students who studied online during the COVID-19 pandemic, the lower frequency of socializing outside to some extent reduces the space and channels for releasing stress, thus increasing the perception of stress, anxiety, and other negative emotions. For these international students, instead of staying in one environment for a long time, going out and socializing with friends to get outside support is an effective way to relieve and vent stress. Previous studies have suggested that social support may reduce genetic and environmental vulnerability and imbue resilience to stress through its effects on the hypothalamic–pituitary–adrenal cortex (HPA) system, the norepinephrine system, and the central oxytocin pathway, so social support is essential for maintaining physical and mental health (Ozbay et al., 2007). Conversely, those international students with low frequency of socialization, lack of social support, and long-term exposure to the same environment cannot relieve the stress from online learning, thus increasing anxiety. Compared with the frequency of socialization, the difference in daily sleep duration, the frequency of physical exercise, and daily study duration was not significantly correlated with anxiety. Our result also suggested that the improving lifestyles, including sleep duration, physical exercise, and study duration, was not as effective as increasing the frequency of socialization in reducing anxiety. Our study is partially consistent with a Malaysian study of Malaysian university students (Mohamad et al., 2021). Our research suggested that active participation in social activities can reduce anxiety in students. The difference is that local studies in Malaysia have shown that sleep quality and BMI were the influencing factors of anxiety. In contrast, in our study, there was no correlation between sleep and BMI and anxiety among international students. On the one hand, this may be due to sample selection bias. On the other hand, it also indicated that increasing social frequency could be more significant for international students in alleviating anxiety than for local students. Therefore, international students should increase the frequency of social activities as possible, instead of staying in the same environment every day, to relieve anxiety.

### Expected Teaching Mode

In this study, the expectation that the teaching mode of the coming semesters (2021 fall & 2022 Spring) would be purely online was a risk factor for anxiety of these international students. Notably, students expecting purely online teaching were more anxious, suggesting that the main factor causing anxiety among these students is not the online learning themselves but the risk of COVID-19 infection brought by face-to-face classes. Compared with those who expected pure face-to-face teaching and online and offline dual-track teaching, international students who expected purely online teaching may be more worried about their health risks caused by the COVID-19 pandemic due to some reasons, such as poor physical fitness or other psychological problems. That is because online classes, compared with offline courses, provide a platform for international students to avoid the risk of infection. In the coming semesters, many schools have announced the resumption and mandatory offline classes, which may increase the anxiety of these students. Therefore, the school had better make special teaching arrangements, such as online and offline dual-track teaching, for those who cannot participate in offline teaching for various reasons rather than forcing completely face-to-face teaching to relieve their anxiety.

### Subjective Experience of Online Learning

In this study, poor subjective experience of online learning (rating the subjective experience of online courses less than 6 out of 10) was a risk factor for anxiety of these international students. Among the international students who studied online during the COVID-19 pandemic, some were upset due to various inconveniences from online classes, such as poor Internet connection and the inability to interact with instructors immediately. These international students may be anxious about their grades or the recognition of their qualifications gained through online teaching by future employers due to poor experiences of online learning. In contrast, international students who rated the experience of online learning higher than 6—those who were more adaptable to online learning—were less likely to experience anxiety. Moreover, there may be a correlation between psychological stress, coping style, adaptability, and mental health (Zimmermann et al., 2012; Zhou et al., 2017). International students with pessimism or anxiety are also more likely to experience poor online teaching.

On the contrary, students with solid adaptability tend to have a better experience of online learning. Our result is also consistent with previous studies that whether students adapt to online courses significantly affects anxiety (Zhao et al., 2021). In addition, people with positive coping styles have better mental health than those with negative coping styles (Wu et al., 2020). Students with high psychological resilience can better understand the meaning of positive coping styles, thus effectively overcoming difficulties in adversity (Hartley, 2011). Therefore, schools should continue to upgrade the facilities for online learning and take other measures to improve the online teaching experience. At the same time, schools can also increase communication with students, genuinely understand the factors that affect students’ online teaching experience and make appropriate corrections. While improving students’ experience of online courses, it is also possible to improve students’ learning efficiency, enthusiasm, academic performance, and mental health. During the COVID-19 pandemic, students with anxiety should also seek outside support during online study, such as reaching out to instructors or seeking psychological counseling (Ozbay et al., 2007).

## Limitations

Some limitations should be acknowledged. Firstly, data were collected through online questionnaires on social platforms, leading to information bias and misclassification. Participants may not have provided accurate information, either included in the study or quickly completed the survey. Therefore, it is necessary to clean and filter the collected questionnaires, check the consistency and logicality of the answers, and adjust invalid and missing values. Due to the international students who participated in this study from the Internet, they did not complete it face-to-face. Secondly, due to the groups to which the questionnaire was collected, the number of international students in each region was not evenly distributed, hence affecting the overall results.

## Conclusion

In conclusion, younger age, living in big cities, low frequency of socialization, the expectation of purely online teaching, and poor subjective experience of online courses were the risk factors for anxiety of these international students who studied online during the COVID-19 pandemic. To minimize the negative impacts of these risk factors of international students who studied online requires the joint efforts of the school and students. The school should provide psychological assistance to the corresponding groups in time and continuously upgrade the facilities to provide a better experience for online learning. At the same time, students should socialize and communicate more with their friends, trying to get more external support to relieve anxiety and stress.

## Data Availability Statement

The original contributions presented in the study are included in the article/supplementary material, further inquiries can be directed to the corresponding author.

## Ethics Statement

The studies involving human participants were reviewed and approved by Medical Ethics Committee, Xiangya Hospital, Central South University. The patients/participants provided their written informed consent to participate in this study.

## Author Contributions

JK, YT, and ZW were responsible for the study design. YT was responsible for collecting the data. YT and JK were responsible for the explanation of the data, data analysis, and drafting the manuscript. XQ, YL, LP, YG, SL, JD, QT, JW, XP, JL, and MS were responsible for the revision of the manuscript. All authors contributed to the article and approved the submitted version.

## Funding

This research was supported by Education Reform Research Project of Central South University, No. 2020JY165.

## Conflict of Interest

The authors declare that the research was conducted in the absence of any commercial or financial relationships that could be construed as a potential conflict of interest.

## Publisher’s Note

All claims expressed in this article are solely those of the authors and do not necessarily represent those of their affiliated organizations, or those of the publisher, the editors and the reviewers. Any product that may be evaluated in this article, or claim that may be made by its manufacturer, is not guaranteed or endorsed by the publisher.

## Abbreviations

GAD-7: Generalized Anxiety Disorder 7-item
OR: Odds ratio
